# CAR T-Cell Therapies in Italy: Patient Access Barriers and Recommendations for Health System Solutions

**DOI:** 10.3389/fphar.2022.915342

**Published:** 2022-06-23

**Authors:** Claudio Jommi, Stefania Bramanti, Marcello Pani, Alessandro Ghirardini, Armando Santoro

**Affiliations:** ^1^ CERGAS (Centre for Research on Health and Social Care Management), SDA Bocconi School of Management), Bocconi University, Milan, Italy; ^2^ IRCCS Humanitas Research Hospital- Humanitas Cancer Center, Rozzano, Italy; ^3^ Agostino Gemelli University Policlinic, Rome, Italy; ^4^ Istituto Superiore di Sanità, Rome, Italy; ^5^ National Center for Telemedicine and New Assistive Technologies, National Institute of Health, Rome, Italy; ^6^ Humanitas University Pieve Emanuele, Milan, Italy

**Keywords:** CAR T-cell therapy, DLBCL, patient access, health system governance, health policy, Italy

## Abstract

CAR T-cell therapy has emerged as a potentially transformative immunotherapy for certain B-cell malignancies including relapsed/refractory diffuse large B-cell lymphoma (DLBCL). Unhindered and appropriate access for eligible patients is essential to enable optimal outcomes and depends on effective interplay of stakeholders and processes along the patient’s therapeutic journey. In Italy, CAR T-cell therapies have been awarded innovation status by the Italian Medicines Agency (AIFA) and were integrated into routine patient care under specific criteria. However, our analysis indicates that fewer than one in five DLBCL patients eligible under the EMA authorization, or around one in three DLBCL patients eligible under the AIFA criteria, received treatment with a licensed CAR T-cell therapy product in 2020. This publication describes key patient access barriers to CAR T-cell therapies in Italy and provides recommendations on potential solutions at the health system level.

## Introduction

### CAR T-Cell Technology in Cancer Therapy

In recent years, there has been a paradigm shift in cancer treatment with the advent of effective immunotherapies that engage the patient’s immune system in the fight against the malignancy (Waldman et al., 2020). CAR T-cell therapies have garnered excitement due to observed response rates in certain hematological malignancies, where they constitute potent new cancer immunotherapy options with potential for long-term patient survival (Abramson et al., 2021; Adami and Maher, 2021; Jacobson et al., 2021; Schuster et al., 2021). CAR T-cells are genetically engineered *ex vivo* from the patient’s immune T-cells to express chimeric antigen receptors (CARs) that are designed to bind to selected tumor antigens. When infused back into the patient, these CAR T-cells will target tumor cells carrying the right antigen for T-cell mediated killing (Waldman et al., 2020).

The first CAR T-cell therapies targeting the B-lymphocyte antigen CD19 (tisagenlecleucel and axicabtagene ciloleucel) were approved in 2017 by the U.S. Food and Drug Administration (FDA) and in 2018 by the European Medicines Agency (EMA) (EMA, 2021b, 2021d; FDA, 2021c, 2022c). In Europe, the following three initial indications received marketing authorization for CAR T-cell therapy in 2018: for relapsed/refractory DLBCL after two lines of systemic therapy (tisagenlecleucel and axicabtagene ciloleucel); for primary mediastinal large B-cell lymphoma (PMBCL) after two lines of systemic therapy (axicabtagene ciloleucel); and for relapsed/refractory B-cell acute lymphoblastic leukemia (ALL) after two lines of therapy or post-stem cell transplant in patients up to 25 years (tisagenlecleucel) (Table 1) (EMA, 2021b; 2021d).

**TABLE 1:** Commercial CAR T-cell therapies approved by the EMA and FDA (May 2022).

Commercial CAR T-cell therapy	Target	Indication	Date of EMA marketing authorization	Date of FDA marketing authorization
tisagenlecleucel/Kymriah^®^	CD19	Paediatric 3L+ ALL	September 2018 (EMA, 2021b)	August 2017 (FDA, 2021c)
		3L+ DLBCL	September 2018 (EMA, 2021b)	May 2018 (FDA, 2021c)
		3L+ HGBL	—	May 2018 (FDA, 2021c)
		3L+ DLBCL from FL	—	May 2018 (FDA, 2021c)
		3L+ FL	March 2022/positive CHMP opinion received (EMA, 2022c)	—
axicabtagene ciloleucel/Yescarta^®^	CD19	3L+ DLBCL	September 2018 (EMA, 2021d)	October 2017 (FDA, 2022c)
		2L+ DLBCL	—	April 2022 (FDA, 2022b)
		3L+ PMBCL	September 2018 (EMA, 2021d)	October 2017 (FDA, 2022c)
		3L+ HGBL	—	October 2017 (FDA, 2022c)
		3L+ DLBCL from FL	—	October 2017 (FDA, 2022c)
		4L+ FL (EMA)	April 2022/positive CHMP opinion received (EMA, 2022d)	April 2021 (FDA, 2022c)
		3L+ FL (FDA)		
brexucabtagene autoleucel/Tecartus^®^	CD19	3L+ MCL (EMA)	December 2020 (EMA, 2021c)	July 2020 (FDA, 2021d)
		2L+ MCL (FDA)		
		Adult 2L+ ALL	—	October 2021 (FDA, 2021d)
isocabtagene maraleucel/Breyanzi^®^	CD19	3L+ DLBCL	April 2022 (EMA, 2022a)	February 2021 (FDA, 2021b)
		3L+ PMBCL	April 2022 (EMA, 2022a)	February 2021 (FDA, 2021b)
		3L+ HGBL	—	February 2021 (FDA, 2021b)
		3L+ DLBCL from FL	—	February 2021 (FDA, 2021b)
		3L+ FL (grade 3B)	April 2022 (EMA, 2022a)	February 2021 (FDA, 2021b)
idecabtagene vicleucel/Abecma^®^	BCMA	4L+ MM (EMA)	August 2021 (EMA, 2021a)	March 2021 (FDA, 2021a)
		5L+ MM (FDA)		
ciltacabtagene autoleucel/Carvykti^®^	BCMA	4L+ MM (EMA)	March 2022/positive CHMP opinion received (EMA, 2022b)	February 2022 (FDA, 2022a)
		5L+ MM (FDA)		

2L+, second or later-line systemic therapy; 3L+, third or later-line systemic therapy; 4L+, fourth or later-line systemic therapy; 5L+, fifth or later-line systemic therapy; CD19, B-lymphocyte antigen CD19 (Cluster of Differentiation 19); BCMA, B-cell maturation antigen; ALL, acute lymphoblastic leukaemia; DLBCL, diffuse large B-cell lymphoma; HGBL, high-grade B-cell lymphoma; FL, follicular lymphoma; PMBCL, primary mediastinal large B-cell lymphoma; MCL, mantle cell lymphoma; MM, multiple myeloma.

DLBCL is the most common of these initial CAR T-cell therapy indications, and accounts for up to 45% of non-Hodgkin lymphomas globally, which corresponds to an estimated DLBCL incidence of 8.2 per 100,000 people in the EU-27 countries (ECIS, 2020; Wild et al., 2020). Relapsed/refractory DLBCL has traditionally been associated with very poor outcomes with no curative treatment options for the majority of the patients. In 2017, an international, multicohort retrospective study reported that only 20% of refractory DLBCL patients would survive beyond two years with standard of care second-line therapy (Crump et al., 2017). Since then, results of separate long-term CAR T clinical trials have been published (Abramson et al., 2021; Jacobson et al., 2021; Schuster et al., 2021), reporting a 4-year overall survival rate of 44% in relapsed/refractory DLBCL patients treated in third line with a CAR T-cell therapy (Jacobson et al., 2021). Also, three independent randomized studies of CAR T-cell therapies in DLBCL patients who relapsed after first-line therapy were conducted, with two of the studies demonstrating improved outcomes in comparison to the standard of care second-line therapy (Bishop et al., 2021; Kamdar et al., 2021; Locke et al., 2022).

By May 2022, six commercial CAR T-cell therapies have received marketing authorization by the FDA or EMA (Table 1) and many more are expected to become established in cancer therapy practice across different types of malignancies in the coming years (in 2021, more than 150 CAR T-cell clinical trials were ongoing) (Adami and Maher, 2021). Alternative CAR therapy approaches are also being investigated, such as CAR therapies using natural killer (NK) cells or allogeneic cell donors that would promise “off-the-shelf” manufacturing capability (Adami and Maher, 2021).

### Usage Setting for DLBCL CAR T-Cell Therapies in Italy

In 2019, the two CAR T-cell therapies for DLBCL (tisagenlecleucel and axicabtagene ciloleucel) were awarded innovation status by the Italian Medicines Agency, AIFA (Agenzia Italiana del Farmaco) (AIFA, 2019a; 2019b). As a result, both CAR T-cell therapies were automatically included on the regional formularies and funded through the national Fondo per i Farmaci Innovativi Oncologici (national fund for innovative oncology drugs), in theory to provide immediate and equal access to eligible patients in Italy (AIFA, 2021a; Jommi and Minghetti, 2015). To account for uncertainties with long-term real-world effectiveness, AIFA, which also is in charge of the negotiation of drug prices, reimbursement and managed entry agreements (Jommi and Minghetti, 2015), defined the first “payment at results” model for these CAR T-cell therapies, with three separate instalments depending on pre-specified endpoints during a 12-month period (AIFA, 2019a; 2019b).

Due to their innovative nature and the complexity of their management, integration of CAR T-cell therapies was tightly controlled by AIFA. CAR T-cell therapies must be provided by centers authorized by the regions, which fulfil the minimal requirements from AIFA, namely: Certification by the National Transplant Center in line with EU directives; JACIE accreditation for allogenic transplantation including clinical, cell collection and cell processing units; availability of an intensive care and reanimation unit; and presence of a multi-disciplinary team adequate for management of the therapy and possible adverse events (AIFA, 2019a; 2019b).

For reimbursement by Italy’s National Health Service (Servizio Sanitario Nazionale), authorized CAR T centers must collect data in an online AIFA drug registry, confirming patient eligibility and appropriateness of treatment along AIFA criteria, with regular follow up assessments of the patient (AIFA, 2019c; 2021c). The key DLBCL patient eligibility criteria for CAR T-cell therapies as stipulated by AIFA can be found in Table 2 and are informed by the registrational trial inclusion/exclusion criteria. Of note, Italy is the only country know to have defined an upper age limit (70 years) for CAR T-cell therapies in DLBCL patients (defined in 2019 and increased to 75 years in May 2022 for tisagenlecleucel (Di Rocco et al., 2021; AIFA, 2022) (Table 2). Advanced age might however not be a predictive factor of fitness for CAR T-cell therapy as indicated by an analysis by Di Rocco et al., 2021, which reported no statistical difference in terms of median overall survival (OS) duration between DLBCL patients below and over 70 years that were considered otherwise eligible for CAR T-cell therapy (Di Rocco et al., 2021). Moreover, several analyses using post-approval, real-world CAR T-cell therapy data from the US CAR-T Cell Consortium and the Center for International Blood and Marrow Transplant Research Cellular Therapy Registry report similar efficacy to the registrational trials despite including an older and broader relapsed/refractory DLBCL patient population with more comorbidities (more than 50% of the patients would not have met the registrational trial eligibility criteria) (Myers et al., 2021).

**TABLE 2:** CAR T-cell therapy eligibility criteria for DLBCL patients.

Key eligibility criteria	AIFA (AIFA, 2019c, 2021c)	Registrational trials (ZUMA-1/JULIET) – combined criteria (ClinicalTrials.gov, 2021a, 2021b)	EBMT recommandation (Yakoub-Agha et al., 2020)
Age	18–70*	≥ 18	No upper age limit
	*defined in 2019. Note that since May 2022 the AIFA age limit has been increased to 75 years for tisagenlecleucel (AIFA, 2022)		
ECOG status	0–1	0–1	0–2; >2 not recommended
Life expectancy	≥ 12 weeks	≥ 12 weeks	Not specified
Prior treatments	Excluded in case of	Excluded in case of	Not a contraindication
	- Prior allogeneic stem cell transplantation	- Prior allogeneic stem cell transplantation	
	- Prior anti-CD19 therapy and absent CD19 expression	- Prior anti-CD19 therapy, anti-CD19/anti-CD3 therapy, gene therapy	
History of auto immune diseases/systemic immunosuppressive treatment	Excluded in case of history of autoimmune disease resulting in end-organ injury or requiring systemic immunosuppression or disease-modifying agents in the last 2 years	Any immunosuppressive medication must be stopped	Not recommended in active autoimmune disease resulting in end-organ injury or requiring systemic immunosuppression of disease-modifying agents in the last 2 years
History of CNS malignancies/diseases	Excluded in case of	Excluded in case of	Individualized risk-benefit assessment
	- Malignancy with CNS involvement	- Malignancy with CNS involvement	
	- Active inflammatory or autoimmune neurological disorders	- Active inflammatory or autoimmune neurological disorders	
	- Other CNS pathologies	- Other CNS pathologies	
Fungal, bacterial, viral, or other infection	Excluded in case of	Excluded in case of: Active HBV/HCV infection - HIV positive	Individualized risk-benefit assessment
	- Active HBV/HCV infection	- Clinically significant active infection	
	- HIV positive	- Currently receiving IV antibiotics	
Adequate renal, hepatic, pulmonary, and cardiac function	Specific parameters to be met	Specific parameters to be met	Specific parameters to be met
Adequate bone marrow reserves (neutrophil, lymphocyte, platelets, hemoglobin count)	Specific parameters to be met	Specific parameters to be met	Specific parameters to be met

Note that additional specific criteria apply.

As with other aggressive malignancies, timely, appropriate and unhindered access to treatment is critical to optimize outcomes for eligible patients with relapsed/refractory DLBCL. The DLBCL patient’s therapeutic journey from diagnosis to CAR T-cell therapy includes several critical steps, which depend on optimal interplay of all stakeholders and processes involved. After diagnosis, DLBCL patients are most commonly treated with the immunochemotherapy combination R-CHOP as first-line treatment. However, up to half of DLBCL patients will be refractory to first-line therapy or will experience relapse. Standard of care in second-line consists of salvage therapy with eligible patients undergoing subsequent autologous stem cell transplantation (Crump et al., 2017). If a DLBCL patient is relapsed or refractory after second-line treatment, the treating physician may consider the patient eligible for CAR T-cell therapy, based on the EMA approved indication and AIFA eligibility criteria. The physician will consult a CAR T center to assess the patient’s eligibility and define the next procedural steps. Laboratory tests and imaging will be conducted to ensure the patient meets the defined criteria: the availability of integrated telemedicine services can facilitate the management of this crucial path. Once the patient has agreed to a CAR T-cell therapy, the patient will be referred and must travel to the CAR T center for the various steps involved in the treatment. Firstly, the patient must undergo leukapheresis to collect peripheral blood mononuclear cells from the blood, which are then shipped to the manufacturing facility for CAR T-cell engineering and expansion. Before re-infusion of the CAR T-cells, the patient often receives bridging therapy for disease control. All patients receive lymphodepleting conditioning chemotherapy to create an optimal environment for the CAR T-cells to expand. After infusion of the CAR T-cells, the patient should remain hospitalized for 10–14 days and for another two to three weeks in the vicinity of the CAR T center to ensure appropriate monitoring post-infusion. Hospitalization time required for CAR T-cell therapies could be reduced in the future with increased real-world experience and potential outpatient administration protocols (Myers et al., 2021). The patient is generally followed up long-term by the referring center (AIFA, 2019c; Yakoub-Agha et al., 2020; AIFA, 2021c).

## DLBCL CAR T-Cell Therapy Access Situation and Barriers in Italy

### Analysis of 2020 DLBCL Patient Access to CAR T-Cell Therapy in Italy

**FIGURE 1:**
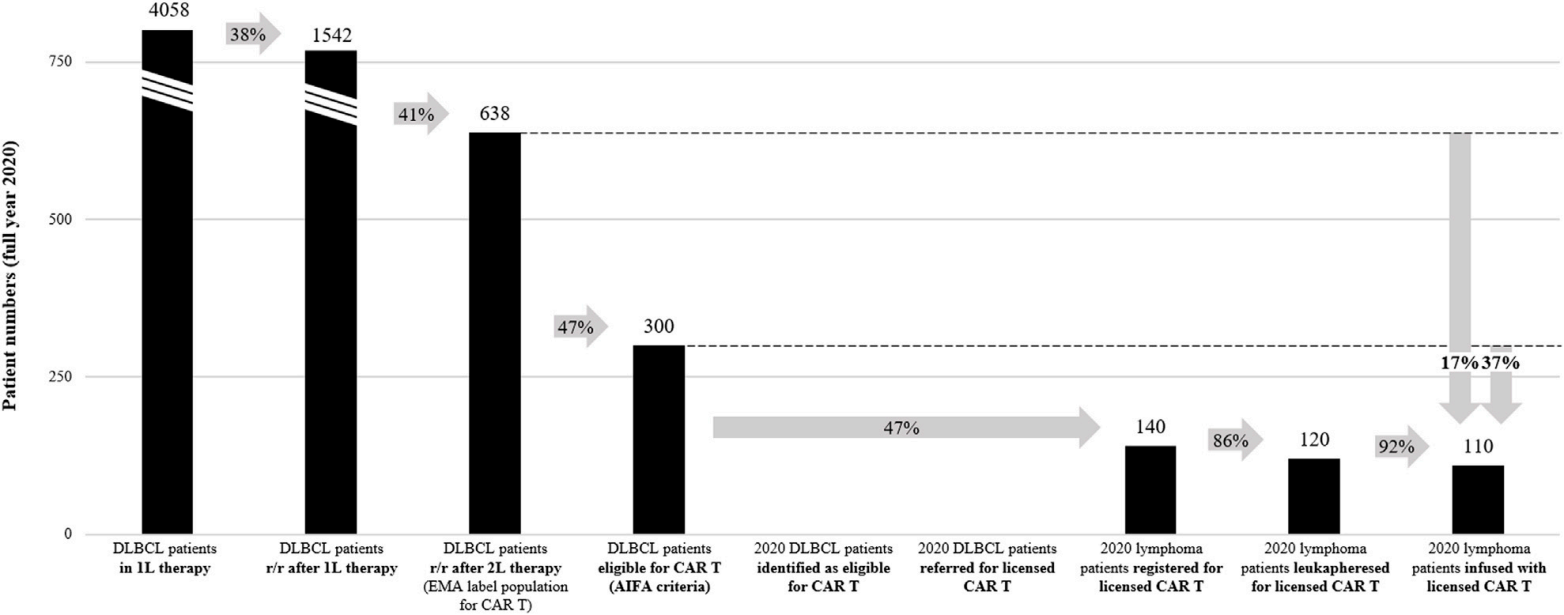
2020 DLBCL CAR T-cell therapy access analysis. Numbers in graph indicate the total patient numbers in Italy in 2020. Arrows indicate percentage of patients arriving at a specific step of the patient journey relative to a previous step. *See* Supplementary Table S1 for calculations and references for the analysis. r/r, relapsed / refractory; 1L, first-line; 2L, second-line.

To examine patient access to CAR T-cell therapies in Italy and to uncover potential systemic barriers, an analysis was conducted of year 2020 DLBCL patient numbers along the key steps of their therapeutic journey, from DLBCL diagnosis to CAR T-cell therapy infusion (Figure 1 and Supplementary Table S1). The analysis used publicly available data to estimate the number of DLBCL patients that underwent systemic lines of therapy and that were theoretically eligible for receiving licensed CAR T-cell therapies. No publicly available data on the number of DLBCL patients treated with licensed CAR T-cell therapies could be identified for the full year 2020 (January to December). Published information from the AIFA registry as well as a multi-center observational study by the Italian Society of Hematology (SIE) report only cumulative data on patient numbers from the date of AIFA registry opening (August 2019 to December 2020) (AIFA, 2021b) or from SIE study initiation (March 2019 to June 2021, respectively (Chiappella et al., 2021). This data therefore would not be supportive of a direct comparison with the epidemiological estimations of DLBCL patients eligible for a licensed CAR T-cell therapy in the year 2020, and would potentially introduce an element of bias, considering the year 2019 ramp up phase when only few CAR T centers were authorized and qualified. Instead, data and estimations for the total number of licensed DLBCL CAR T-cell therapies in Italy in the year 2020 (tisagenlecleucel and axicabtagene ciloleucel aggregated) as provided by Kite Pharma Inc./Gilead Sciences S.r.l in personal communication with the authors were used for the analysis (Kite/Gilead, 2021). To ensure robustness of the analysis, a cross-check of the different data sets (Kite/Gilead, AIFA registry, SIE study) using the average number of DLBCL CAR T-cell therapies per month was performed (*see* Supplementary Table S1 for data and calculations underlying the analysis).

Based on the 2015 AIRTUM Italian cancer figures, there were an estimated 4,559 new cases of DLBCL in Italy in 2020 (Supplementary Table S1) (AIRTUM Working Group, 2016). This corresponds to an incidence rate of 7.6 per 100,000 inhabitants, which is comparable to other estimations for Italy (Heine et al., 2021). Of these DLBCL patients, 4,058 (89%) are estimated to have received first-line therapy (Belleudi et al., 2021), and 1,542 (34%) to have been treated in second-line after relapse or being refractory to first-line therapy. 638 DLBCL patients (14%) are estimated to have relapsed or have been refractory after two lines of systemic therapy, which corresponds to the EMA-approved indication for CAR T-cell therapies (Belleudi et al., 2021; Di Rocco et al., 2021). Of note, other estimations for Italy (Heine et al., 2021) as well as from the French Haute Autorité de Santé (HAS, 2021b; 2021a) report a higher percentage (17%) of DLBCL patients relapsed or refractory after two lines of systemic therapy, which would correspond to 775 Italian patients in 2020. For centers to receive reimbursement for CAR T-cell therapies provided, specific patient selection criteria defined by AIFA and based on the registrational trial inclusion/exclusion criteria must be applied (Table 2) (AIFA, 2019c; 2021c). Under these criteria, it was estimated that only 47% of the EMA-approved DLBCL population would be eligible for CAR T-cell therapy in Italy (Di Rocco et al., 2021), corresponding to 300 patients in 2020 (7% of the entire DLBCL population). Interestingly, the French Haute Autorité de Santé estimated that based on the findings from the registrational trials, 60% of the EMA-approved third-line DLBCL population would have the health status and life expectancy required to undergo CAR T-cell therapy (HAS, 2021a; 2021b). In Italy, this would correspond to 383 patients in 2020 (60% of 638 DLBCL patients relapsed or refractory after two lines of therapy).

Based on available data for the full year 2020 provided by Kite Pharma Inc./Gilead Sciences S.r.l in personal communication (Kite/Gilead, 2021), this analysis estimates that in 2020, 140 patients were approved for licensed CAR T-cell therapy in Italy (47% of the eligible DLBCL population under AIFA criteria), 120 patients underwent leukapheresis, and 110 patients were treated with a licensed CAR T-cell therapy (on average nine patients per month, 37% of the eligible DLBCL population under AIFA criteria and 17% of the EMA-approved DLBCL population). The median duration from approval of patient eligibility (registration in AIFA registry) to CAR T-cell therapy was 63 days (AIFA, 2021b). No information was available on the number of DLBCL patients identified and referred for CAR T-cell therapy, or the duration from patient identification to approval of patient eligibility. The above estimation of the DLBCL CAR T-cell therapy rate is within range of data reported by the AIFA registry (on average eight patients per month, 137 DLBCL patients treated between August 2019 and December 2020) (AIFA, 2021b). In contrast, the SIE observational study reports a lower DLBCL CAR T-cell therapy rate (on average seven patients per month, 191 patients treated between March 2019 and June 2021) (Chiappella et al., 2021), potentially related to covering a longer timeframe and not the same dataset than the AIFA registry and this analysis. Of note, it is likely that fewer DLBCL patients had access to licensed CAR T-cell therapies in 2020 than reported in this analysis, as the datasets also included other lymphoma subtypes besides DLBCL (for instance the AIFA registry and SIE observational study report 10% and 18% PMBCL patients, respectively) (AIFA, 2021b; Chiappella et al., 2021).

This analysis indicates that in 2020, around 190 Italian DLBCL patients were not treated with a licensed CAR T-cell therapy despite being eligible under the criteria defined by AIFA. Significant barriers limiting patient access could exist, especially in the steps before treatment decision in the CAR T centers (only 140 of 300 theoretically eligible patients under AIFA criteria received approval for a licensed CAR T-cell therapies in 2020). It is important to acknowledge limitations of the current analysis. While CAR T-cell therapy units generally continued to operate in 2020 despite national lockdown, it is likely that the COVID-19 pandemic might have had a negative impact on the number of patients that were seeking and given access to CAR T-cell therapy (In Salute News, 2020). Also, as many CAR T centers only became operational in late 2019 and early 2020, a potential learning curve is not represented in the aggregated full year 2020 analysis. Finally, a fraction of CAR T-cell therapy eligible patients may have been accepted into clinical trials or early access programs instead of receiving a licensed CAR T-cell therapy in 2020. For a more comprehensive understanding of the CAR T-cell therapy access situation across Italy, in particular at region or network level, systematic data collection along the journey of a DLBCL patient would be required.

### Barriers to Access of CAR T-Cell Therapy for DLBCL Patients in Italy

Based on the analysis conducted and professional expertise, the authors have identified a number of core barriers to access of CAR T-cell therapy for DLBCL patients in Italy. The following views on CAR T access barriers, best practices, and health system and policy recommendations were collected through individual, structured phone interviews with the authors, who were selected on the grounds of their expertise and interest in contributing to this publication (sample of convenience) (Supplementary Table S2) (Lohr, 2010). The interview findings included in this analysis represent the authors’ validated collective personal opinions.

#### Patient Identification Outside of a CAR T Center

Because DLBCL can be a rapidly progressing disease, the identification of a CAR T-eligible patient must happen early and correctly to ensure optimal outcomes. Already one month wait time for CAR T-cell therapy was calculated to increase the relative mortality rate by 6.2% after one year (Tully et al., 2019). Also, the SIE observational study reports that 5% of 208 already leukapheresed patients in Italy did not continue to receive CAR T-cell therapy due to a rapid disease progression and worsening of their clinical condition (Chiappella et al., 2021), highlighting the urgency of timely access to CAR T-cell therapy. However, many patients referred to Italian CAR T centers are already at an advanced stage, with multiple lines of prior therapy and reduced health status, which can limit their fitness and eligibility to receive CAR T-cell therapy. According to AIFA data, 42% of DLBCL patients registered for a licensed CAR T-cell therapy received three or more lines of prior therapy (29% with three lines, 13% with more than four lines) (AIFA, 2021b), when patients would be eligible after a relapse from second-line therapy. Also, the authors’ perception is that fewer potential eligible patients arrive in CAR T centers than expected based on epidemiological estimations, indicating challenges with identification or referral of DLBCL patients for CAR T-cell therapies.

Reasons for a suboptimal identification of eligible DLBCL patients may include limited understanding of these novel cell therapies by patients and haemato-oncologists outside of CAR T centers, especially in respect to their potential benefits, safety profiles, eligibility criteria and the importance of early patient identification. Particularly for small peripheral centers that see many types of malignancies, there may be limited practical knowledge in correctly identifying CAR T-eligible DLBCL patients. Situations where haemato-oncologists are only weakly integrated in regional onco-hematological care networks might also impede timely and correct identification of patients, as support from the network (for instance through tumor boards providing eligibility guidance) would not be accessible.

Existing best practices in Italy to support early patient identification include primarily educational activities. For instance, the Humanitas Cancer Center has successfully organized virtual roadshow events directly with referring centers to increase knowledge on CAR T-cell therapy and optimal patient selection.

#### Patient Referral to a CAR T Center

Patient referral to specialized CAR T centers can be a complex and time-consuming process, adding significant burden to healthcare professionals and patients. Management of patient referral, particularly across regions, was reported as a major challenge in a survey with different Italian CAR T and ATMP centers (ATMP Forum, 2021). Referral distances can be considerable and may even require patients to travel outside of their own region. As DLBCL patients with a reduced health status often require assistance, family members are also likely required to travel with them. And in case the referral process is not executed optimally it can delay access to CAR T-cell therapy and impact the overall outcomes of patients. Regional inequalities for CAR T-cell therapy access appear to exist in Italy: AIFA registry data from August 2019 to December 2020 indicates that DLBCL patients from only 10 out of 20 regions were treated with licensed CAR T-cell therapies (AIFA, 2021b).

The authors see reasons for suboptimal patient referral in the lack of formalized networks for CAR T patient referral and the absence of nationally harmonized referral standards. Referral often occurs through personal contacts or individual center-to-center agreements that do not necessarily take patient travel burden or waiting time into account (ATMP Forum, 2020). Also, referring centers are not always strongly integrated into the overall CAR T-cell therapy journey of a DLBCL patient (for instance, there is no designated physician with “CAR T-cell therapy responsibility” in referring centers), which can result in suboptimal referral collaboration and processes. Moreover, CAR T centers are so far not evenly distributed in Italy, due to regionalized decision-making on center selection and delays in center authorization and qualification by regional authorities and pharmaceutical manufacturers. In December 2020, 70% of qualified CAR T centers were concentrated in only four regions (Lazio, Lombardy, Piedmont, Tuscany) (AIFA, 2021b). This has created situations where referral must happen over large distances, increasing complexity and logistical barriers.

Best practices for optimal referral collaboration and processes for CAR T-cell therapies exist. For instance, certain Italian regions already have implemented strong hematological referral networks with specified treating and peripheral referring centers that are also used for CAR T-cell therapy referral. The Emilia-Romagna region has established a regional network including centralized decision making on patient eligibility and waiting list for CAR T-cell therapies (ATMP Forum, 2020; In Salute News, 2020). Also, integrated care pathways defined at regional or local level (so-called Percorsi Diagnostici Terapeutici Assistenziali) are used by certain regions to ensure quality and harmonized processes for CAR T-cell therapies (ATMP Forum, 2020; In Salute News, 2020). Finally, other countries such as Netherlands and the UK have established national clinical CAR T boards, which confirm patient eligibility at the national level and allocate patients to suited CAR T centers, based on urgency and center capacity considerations (NICE, 2019; HOVON, 2022).

#### Pre-treatment Approval of CAR T-Cell Therapy Funding

In addition to confirmation of patient eligibility by the CAR T center based on medical criteria, the center has to ensure that funding for CAR T-cell therapy product and procedure costs will be provided. To ensure product funding, the center must register DLBCL patients intended for a licensed CAR T-cell therapy with an online AIFA registry platform, confirming that the patients meet the AIFA’s eligibility criteria (AIFA, 2019c; 2021c). Conversely, funding of CAR T-cell therapy procedures has to be approved by the regional authorities. Centers are unlikely to proceed with treatment without full approval for product and procedure funding. Any delays in the approval process will also delay access to CAR T-cell therapy and potentially impact the patient’s final outcomes.

The authors identify the following key barriers to ensuring appropriate CAR T-cell therapy funding at the patient level. For out-of-region referred patients, authority approvals in both referring and receiving regions must be given to ensure procedure costs are covered and can be billed to the out-referring region. The approval process can be very time consuming for certain out-referring regions and may result in delays to therapy access for the patient. For instance, some regions rely on ad-hoc contracts with out-referring regions to agree on cost-transfer for CAR T-cell therapy (ATMP Forum, 2020). Complications with cost transfer for out-of-region referrals arise in part also due to a lack of a nationally harmonized tariff for CAR T-cell therapy procedures. Currently, regions use different tariffs to cover CAR T-cell therapy procedures, for instance tariffs for allogeneic or autologous stem cell transplantation, as well as CAR T specific tariffs (ATMP Forum, 2020, 2021). This heterogenous funding situation can create financial challenges for CAR T centers that are paid *via* insufficient tariffs. Furthermore, in some regions, there is informal guidance to not refer patients out-of-region and to avoid redirecting regional health funds to other regions. While this potentially makes sense from a referral travel perspective, this practice should not result in situations where the patient has increased wait time as the regional CAR T center has only limited treatment capacity. Finally, while the cost of CAR T-cell therapies is currently covered through the national Fondo per i Farmaci Innovativi Oncologici, these arrangements are currently set to expire in the second half of 2022 (AIFA, 2021a). After this point, CAR T-cell therapies will be covered directly by regional health formularies and budgets, which will almost certainly create additional challenges and potential approval delays due to their high cost.

While CAR T-cell therapy funding barriers would be best addressed at a systemic level, best practices exist where regional authorities (for instance in Lombardy) ensure decision on funding approval within 24 h and provide CAR T centers in the region with an assurance that procedure costs for out-of-region referred patients will also be covered in the case that the out-referring region would not pay.

#### CAR T-Cell Therapy Delivery in Qualified Treatment Centers

Therapy with a personalized CAR T-cell product is complex and requires specific capabilities and multidisciplinary coordination from professionals at qualified CAR T centers (Yakoub-Agha et al., 2020). In addition to organizational and training needs, significant investment in center infrastructure is often necessary, for instance to expand intensive care unit (ICU) space or to adjust to specific CAR T-cell therapy logistical requirements. In a 2020 survey with different Italian CAR T centers, ensuring quality assurance requirements as well as availability of infrastructures, and specifically infrastructures and personnel for leukapheresis, were ranked as top challenges (ATMP Forum, 2020). The centers also identified specific training needs for CAR T-cell therapy management, for instance in respect to CAR T-cell therapy logistical aspects at the hospital pharmacy (ATMP Forum, 2020). Unless addressed, challenges to sufficiently provide the necessary capabilities, resources and infrastructure can impact the center’s overall capacity to deliver CAR T-cell therapy to DLBCL patients.

The authors see the following key barriers to ensuring optimal CAR T center set up and capacity. Firstly, qualified professionals with critical responsibilities for the patient’s treatment and care are a scarce resource. Also, infrastructural expansion might be limited by the availability of additional space to occupy as well as conflicting priorities between different therapy areas (as exemplified by the COVID-19 pandemic’s high use of ICU beds). Moreover, a lack of adequate planning and support by regional and national authorities for necessary infrastructural and organizational investments can leave CAR T centers carrying investment costs by themselves. This can result in financial sustainability challenges for CAR T centers, amplified in case of insufficient per-patient funding tariff for CAR T-cell therapy procedures.

An example of best practice for ensuring sustainable CAR T-cell therapy delivery at qualified centers is the Careggi University Hospital, which conducted an early assessment of the center’s organizational and resource needs for CAR T-cell therapy in collaboration with the regional health authority in Tuscany, resulting in additional funding for the CAR T center. Another example is in Emilia-Romagna, where the regional authority has followed a focused governance and management approach to CAR T-cell therapy implementation; the Policlinico Sant’Orsola-Malpighi was selected as the region’s reference center for CAR T-cell therapies. To anticipate future capacity challenges due to increasing patient numbers, the regional authority will continuously assess the need for further CAR T centers. In addition, a dedicated expert commission was established to define, evaluate, and optimize CAR T-cell therapy protocols and pathways based on experiences being made (ATMP Forum, 2020; In Salute News, 2020). Finally, CAR T center selection by regional authorities in different regions was supported by newly created working groups on CAR T-cell therapies (ATMP Forum, 2021).

## Recommendations to Address the Identified CAR T-Cell Therapy Access Barriers

Based on the above discussed key CAR T-cell therapy access barriers for DLBCL patients, the authors propose five areas of recommendation to act at the health system level.

### Improve Information and Education to Referring Haemato-Oncologists and Patients

Educational programs and supportive materials for referring haemato-oncologists and DLBCL patients on CAR T-cell therapy eligibility, the therapeutic pathway and clinical results, will play a critical role in improving timely identification and referral of CAR T-cell therapy eligible patients, especially when considering that additional CAR T-cell therapies will become available in the near future. Ideally, such initiatives to support awareness and better understanding of CAR T-cell therapy will be implemented collaboratively by the institutions, the clinical centers, the Italian Society of Hematology, patient groups and the pharmaceutical manufacturers.

### Ensure Data Collection Along the DLBCL Patient Pathway

Systemic collection of DLBCL patient data also in referring centers could support better understanding of the specific barriers along the DLBCL patient journey and potentially improve early identification of high-risk patients who would benefit from rapid access to CAR T-cell therapies. Such data collection, for instance through a national disease-specific registry that would integrate the already existing AIFA registry, should include newly-diagnosed DLBCL patients and potentially eligible CAR T-cell therapy patients and track them along their disease journey. Regular analysis of this data could also inform necessary improvements (for instance planning of CAR T-cell therapy capacity based on regional patient flows/demand) and allow assessment of the impact of such improvements over time.

### Establish Harmonized Standards and Governance for CAR T Referral Networks

National guidelines and governance for CAR T referral networks could help to establish harmonized standards for CAR T clinical networks and referral processes, that are connected with existing onco-hematologic networks and the national rare tumors network. For instance, collaboratively developed by the Italian Society of Hematology, centers, and regional authorities and in alignment with the Istituto Superiore di Sanità, such governance should define processes for information exchange across networks and regions to break silos that currently limit interaction, also across the borders of individual regions. Building on existing best practices at a regional level, guidelines could specify referral pathways within and across regions, identifying treatment and peripheral referring centers for CAR T-cell therapy. Guidelines on referral pathways could also require that capacity and wait times of CAR T centers is taken more strongly into consideration. For instance, information systems could provide visibility of CAR T center capacity/wait times to referring centers and help inform choice of the referral destination. Finally, considerations could be made as to whether experienced referring centers in a network could provide specific steps of the CAR T-cell therapy process, such as patient management before or after CAR T infusion, which could support a more efficient use of existing resources within a network.

### Early and Coordinated Planning for CAR T Centers

National planning and definition of more specific organizational standards for CAR T centers would help ensure that specialized centers for CAR T-cell therapy are established according to population needs and equipped with the necessary resources to provide optimal patient care. Planning should be initiated before marketing authorization and could be based on regular horizon scanning activities in collaboration between national and regional health authorities, to anticipate new requirements and ensure time for selection and authorization of new CAR T centers. Authority support for centers could be linked to their performance (for instance, number of treatments, speed of treatment access, and quality of care, including patient reported outcomes and experiences) to incentivize implementation of quality standards and further improvements. Establishing a coordinated network of excellence centers according to national quality standards and authorization criteria could also represent an important stimulus for regions to develop and improve oncological care and networks further. The early and coordinated planning for CAR T centers should also provide for a clear identification of the role of commercial and non-commercial licensed CAR T-cell therapies. The Italian authorities have provided public funding to develop an academic network for non-commercial CAR T-cell therapies (Gazzetta Ufficiale Della Repubblica Italiana, 2020). Building on this, it could make sense to coordinate public funding and activities to support development of a national network for both commercial and non-commercial licensed CAR T-cell therapies. In the future “off-the-shelf” and local “point-of-care” manufacturing might further increase CAR T-cell therapy availability and reduce overall cost to the health system.

### Sustainable and Equal CAR T-Cell Therapy Funding

CAR T-cell therapies have been approved as innovative treatments by AIFA, and authorities need to ensure that appropriate funding systems are set up to remove financial barriers and support access for all eligible patients. To support financial sustainability of centers, the current CAR T-cell therapy procedure funding should be revised, taking economic impact and organizational requirements for CAR T centers better into consideration. This could be best achieved through defining a specific CAR T-cell therapy procedure tariff at national level, which would likely also reduce barriers with approval of out-of-region referred patients and cost-transfer to the out-referring region. Authorities, besides facilitating rapid cost compensation for cross-regional CAR T patient mobility, also must ensure that innovative high-cost treatments like CAR T-cell therapy remain available to patients also once they transition out of the Fondo per i Farmaci Innovativi Oncologici to regional health formularies and budgets.

## Discussion

Future cell and gene therapy innovation as well as other personalized treatments will likely share many of the challenges discussed here for DLBCL CAR T-cell therapies. A growing number of patients are expected to benefit from such transformative treatments, including future CAR T-cell therapies in new indications, in earlier lines of therapy, and with novel clinical approaches that can further improve efficacy and long-term survival (Adami and Maher, 2021; Ernst et al., 2021). This development, together with the potential need for additional authorized centers providing complex and resource-intense therapies, will undoubtedly increase the pressure on the health system further. It is important to build on the learnings from the initial DLBCL CAR T-cell therapies and act concertedly across health system stakeholders to ensure patient access to future innovative therapies and health system sustainability in the long term. The authors hope that this study can support the process of evolving the Italian health system by creating awareness of the barriers to address and by presenting best practices and health policy recommendations that ultimately allow greater access to essential therapy for patients.
