# Supplementary material for: CAR T-Cell Therapies in Italy: Patient Access Barriers and Recommendations for Health System Solutions
Source: Front Pharmacol. 2022 Jun 23;13:915342. doi: 10.3389/fphar.2022.915342 (PMC9275825; doi:10.3389/fphar.2022.915342)
Supplement: Supplementary file 1 [file Table1.docx]

Supplementary Material

**Supplemental Table 1.** 2020 DLBCL CAR T-cell therapy access analysis

|  | Number | Comments / References |
| --- | --- | --- |
| **DLBCL incidence** | 4559 | - Estimation based on AIRTUM working group incidence data for 2015 in Italy after correction for 2015 to 2020 population change (AIRTUM Working Group et al., 2016; Worldometers, 2021b) - The AIRTUM estimation corresponds to an incidence of 7.6 per 100,000 inhabitants which is comparable to estimations by Heine et al. 2021 (7.5 / 100,000) (Heine et al., 2021a) and incidence estimations for France as published by French Haute Autorité de Santé (7.4 / 100,000) (HAS, 2021a, 2021b; Worldometers, 2021a) |
| **DLBCL patients in first-line systemic therapy** | 4058 | - Estimation based on Belleudi et al. 2021 (89% in first-line therapy within 6 months from DLBCL incidence) (Belleudi et al., 2021) - The estimation for Italy by Belleudi et al. 2021 is comparable to estimations for France as published by French Haute Autorité de Santé (86% of DLBCL patients undergoing first-line systemic therapy) (HAS, 2021b, 2021a) |
| **DLBCL patients in second-line systemic therapy (relapsed / refractory after first-line therapy)** | 1542 | - Estimation based on Belleudi et al. 2021 (38% of first-line treated patients going into second-line therapy) (Belleudi et al., 2021) - The estimation for Italy by Belleudi et al. 2021 is comparable to estimations for France as published by French Haute Autorité de Santé (37% of first-line treated patients going into second-line therapy) (HAS, 2021a, 2021b) |
| **DLBCL patients relapsed / refractory after second-line therapy**  **(EMA DLBCL label indication for licensed CAR T-cell products)** | 638  (-775) | - Estimation for Italy based on Belleudi et al. 2021 and Di Rocco et al. 2021 (14% of DLBCL patients are refractory or in relapse after second-line therapy) (Belleudi et al., 2021; Di Rocco et al., 2021) - Note that the estimations by Belleudi et al. 2021 and Di Rocco et al. 2021 are lower than estimations for Italy by Heine et al. 2021 and for France as published by French Haute Autorité de Santé (17% of DLBCL patients are refractory or in relapse after second-line therapy and are eligible for CAR T-cell therapy, which equals to 775 patients a year in Italy) (HAS, 2021a, 2021b; Heine et al., 2021b) |
| **DLBCL patients eligible for licensed CAR T products under AIFA reimbursement criteria** | 300  (-383) | - Estimation for Italy based on Di Rocco et al. 2021 (47% of refractory / relapsed DLBCL patients after second-line therapy meet CAR T-cell therapy eligibility criteria of AIFA as approximated by the registrational trial JULIET inclusion / exclusion criteria) (Di Rocco et al., 2021) - Note that the estimations by Di Rocco et al. 2021 on the CAR T-cell therapy eligible DLBCL population are lower than estimations published by French Haute Autorité de Santé based on the findings from the registrational trials (60% of the EMA approved DLBCL population are estimated to have the health status and life expectancy required for CAR T-cell therapy, which equals to 383 patients a year in Italy) (HAS, 2021b, 2021a) |
| **DLBCL (and PMBCL) patients registered for licensed CAR T-cell therapy (2020)** | 140 | - Estimation based on data provided by Kite Pharma Inc./ Gilead Sciences S.r.l in personal communication (Kite/Gilead, 2021); corresponds on average to 11.7 patients per month - This estimation is within range of data reported by the AIFA registry (on average 9.7 patients per month, 164 DLBCL patients registered between August 2019 and December 2020) (AIFA, 2021b) |
| **DLBCL (and PMBCL) patients leukapheresed for licensed CAR T-cell therapy (2020)** | 120 | - Estimation based on data provided by Kite Pharma Inc./ Gilead Sciences S.r.l in personal communication (Kite/Gilead, 2021); corresponds on average to 10.0 patients per month - This estimation is higher than data reported by Chiappella et al. 2021 of a multi-center prospective observational study of the Italian Society of Hematology (208 lymphoma patients that have been leukapheresed for CAR T-cell therapies between March 2019 and June 2021, corresponding to 7.4 patients leukapheresed per month) (Chiappella et al., 2021) |
| **DLBCL (and PMBCL) patients infused with licensed CAR T-cell therapy**  **(2020)** | 110 | - Estimation based on data provided by Kite Pharma Inc./ Gilead Sciences S.r.l in personal communication (Kite/Gilead, 2021); corresponds on average to 9.2 patients per month; 92% of patients that have been leukapheresed were infused with a licensed CAR T-cell therapy - This estimation is within range of data reported by the AIFA registry (on average 8.1 patients per month, 137 DLBCL patients treated between August 2019 and December 2020) (AIFA, 2021b) - This estimation is higher than data reported by Chiappella et al. 2021 of a multi-center prospective observational study of the Italian Society of Hematology (191 lymphoma patients that have been infused with CAR T-cell therapies between March 2019 and June 2021, corresponding to 6.8 patients treated per month) (Chiappella et al., 2021)). The rate of leukapheresed patients that undergo CAR T-cell therapy is comparable between this analysis (92%) and data reported by Chiappella et al. 2021 (92%) (Chiappella et al., 2021) - Please note that the lymphoma patients reported to have undergone CAR T-cell therapy in this analysis represent not only DLBCL patients but also PMBCL patients (10% PMBCL patients in AIFA registry and 18% PMBCL patients in study of Italian Society of Hematology) (AIFA, 2021b; Chiappella et al., 2021) |

**Supplemental Table 2.** Interview questions for participating authors

| **No.** | **Interview question** |
| --- | --- |
| **1** | What are your estimations for the number of DLBCL patients in Italy that meet the EMA label requirements; that meet the AIFA eligibility criteria for CAR T-cell therapies? |
| **2** | To your knowledge, what data is available in Italy on the number of DLBCL patients that have undergone treatment with licensed CAR T-cell therapies?  *(e.g., registry data, publications, on the number of DLBCL patients registered/ approved, leukapheresed, infused with CAR T-cell therapies)* |
| **3** | If you consider the journey of a DLBCL patient from diagnosis to CAR T-cell infusion, in which key steps do you identify challenges resulting in delays or in barriers to patient access?  *(e.g., patient identification, patient referral, approval of CAR T-cell therapy funding, delivery of CAR T-cell therapy at qualified treatment centers)* |
| **4** | In your opinion, what are the main challenges for DLBCL patient access to CAR T-cell therapies in Italy? |
| **5** | What do you consider to be the underlying causes of those challenges? |
| **6** | Considering future developments, what do you expect will become the priority challenges for patient access to CAR T-cell therapies (or to cell and gene therapies with a similar challenge profile)? |
| **7** | Do you have examples of existing best practices for ensuring patient access to CAR T-cell therapies that could represent relevant learnings for the health system? |
| **8** | In your opinion, what should be the ambition of the health system in respect to CAR T-cell therapies? |
| **9** | What could be potential health system level solutions and coordinated actions to overcome the identified challenges for patient access to CAR T-cell therapies in Italy? |
